# Neural correlates of Bayesian social belief updating in the medial prefrontal cortex

**DOI:** 10.1093/cercor/bhaf251

**Published:** 2025-09-09

**Authors:** Lieke Hofmans, Wouter van den Bos

**Affiliations:** Department of Developmental Psychology, University of Amsterdam, Nieuwe Achtergracht 129b, 1018 WS Amsterdam, The Netherlands; Motivation, Brain and Behaviour Lab, Paris Brain Institute (ICM), Hôpital de la Pitié-Salpêtrière, 47 bd de l'Hôpital, 75013 Paris, France; Department of Developmental Psychology, University of Amsterdam, Nieuwe Achtergracht 129b, 1018 WS Amsterdam, The Netherlands

**Keywords:** Bayesian decision-making, computational modeling, neuroimaging, social learning, uncertainty

## Abstract

Social learning, a hallmark of human behavior, entails integrating other’s actions or ideas with one’s own. While it can accelerate the learning process by circumventing slow and costly individual trial-and-error learning, its effectiveness depends on knowing when and whose information to use. In this study, we explored how individuals use social information based on their own and others’ levels of uncertainty. Participants performed a social information use task in which they could revise their initial estimate after viewing a peer’s estimate. Uncertainty was manipulated by varying the amount of information provided before their decision and by manipulating the peer’s reported confidence. As expected, adjustments were larger when individuals themselves were less certain and the peer was more confident. Through a combination of Bayesian computational modeling and neuroimaging analyses, we were able to identify regions in the anterior and ventral medial prefrontal cortex where neural activity overlapped in response to lower personal certainty, higher peer confidence, and larger belief updates after viewing the peer’s estimate. We discuss how these regions in the medial prefrontal cortex likely serve as a convergence zone for the preparation and execution of integrating certainty estimates.

## Introduction

Social learning is a hallmark of human behavior and entails the use of others’ information by copying the behavior or ideas of others or integrating them with your own (Bandura et al. 1963). Learning from other people’s errors and rewards circumvents the need for slow and costly individual trial-and-error learning and thereby considerably speeds up the learning process (McElreath et al. 2008; Najar et al. 2020; Morin et al. 2021). However, the usefulness of social information depends on (i) our own current knowledge and (ii) the expertise of the other. First, it is more useful to rely on others in situations in which we are uncertain about what to do (Laland 2004; Kendal et al. 2018; Ciranka and van den Bos 2020). Indeed, previous studies have shown that people search for and use more social information when they are uncertain, not only in the context of perceptual decisions but also in predicting outcomes like US elections (Moutoussis et al. 2016; Olsen et al. 2019; Tump et al. 2020; Gradassi et al. 2022; Slagter et al. 2023). Second, previous studies have shown that people rely more on others when these others are more proficient (Laland 2004; Wood et al. 2013; Kendal et al. 2018; Olsen et al. 2019). To communicate their proficiency or the value of social information, others often indicate their level of confidence. Previous studies have indeed shown that people tend to rely more on others when they report higher confidence (Zarnoth and Sniezek 1997; Tump et al. 2020; Gradassi et al. 2022). Thus, both own certainty and others’ reported confidence are taken into account when deciding to use social information. However, little is known about how personal and social information are integrated at the cognitive level. Here, we will combine an experimental task that manipulates own certainty and others’ reported confidence with computational modeling and neuroimaging to address this question.

**Fig. 1:**
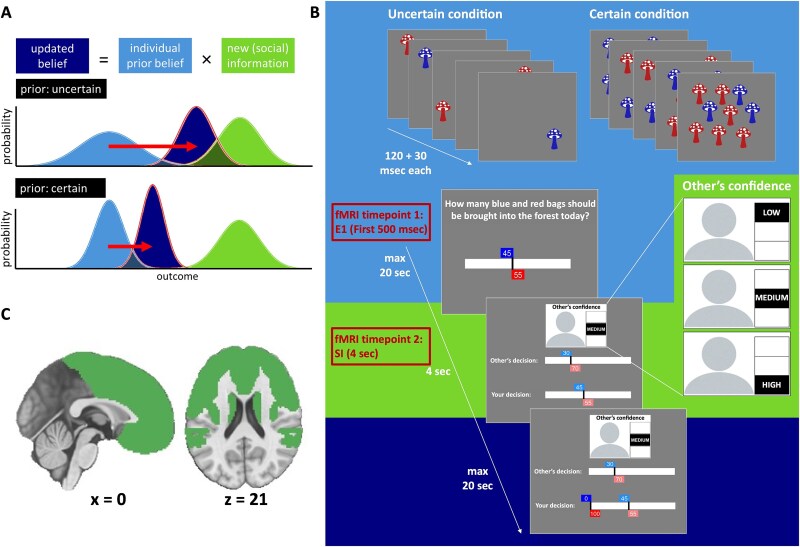
Bayesian updating under uncertainty. A) An agent combines their prior personal belief or information (light blue) with new social information (green) to arrive at an updated belief (dark blue). Higher reliability is represented as a narrower distribution. In the case of high prior uncertainty (top panel), the prior information is assigned a relatively low weight, resulting in a stronger impact of the social information (red arrow). Under certainty (bottom panel), the prior information is assigned a relatively high weight, resulting in a less pronounced impact of the same social information. Similarly, if the social information comes from a peer who reports to be very confident, the social information will be assigned a higher weight than when the peer reports not to be confident, resulting in a higher impact of the social information. B) Task schematic “experiment 2” (fMRI): Participants first view 5 fields of either 5 (uncertain condition) or 45 (certain condition) mushrooms each, based on which they can submit their first estimate using a slider. This constitutes their individual prior belief (light-blue background). The first 500 ms that the participant is prompted to submit their first estimate is our first timepoint for fMRI modeling. They then see the peer’s estimate and associated confidence report, which can be low, medium, or high. This constitutes the social information (green background), which is our second timepoint for fMRI modeling. Based on the social information, they are then prompted to submit their second estimate, which constitutes their belief update (dark-blue background). C)The brain mask used for the fMRI analyses included the frontal lobes, insula, TPJ, and striatum.

Empirical psychological and neuroscience research has established that during decision-making, humans engage in Bayesian precision weighting to capture the uncertainty that accompanies pieces of information by (Bach and Dolan 2012; van Bergen et al. 2015; van Bergen and Jehee 2019; Yon and Frith 2021; Geurts et al. 2022). This entails that more reliable information, including personal information when one is more certain or social information stemming from someone who reports to be very confident, is considered to be more precise and is therefore given greater consideration (Behrens et al. 2008; Perreault et al. 2012; Toelch et al. 2014; Ciranka and van den Bos 2020; Hofmans and van den Bos 2022). Thus, according to a Bayesian model (Fig. 1A), the more reliable a piece of information, the narrower its probability distribution, meaning it is more precise and has a stronger influence on behavior. Conversely, less precise information has a weaker impact on behavior and a broader probability distribution. Although there is ample evidence that people use these Bayesian principles when making decisions (Gershman 2015; Gershman and Uchida 2019; Ciranka and van den Bos 2020; Yon and Frith 2021), it has also been shown that people use heuristics or biases (Kendal et al. 2018; Molleman et al. 2020; Slagter et al. 2023). For example, using a perceptual judgment task, it has been shown that participants combined Bayesian updating in response to social information with a simple heuristic to stay with their own original decisions, without considering the other’s information (Molleman et al. 2020). These studies suggest that at the cognitive level, social learning entails a combination of Bayesian updating and heuristic use.

At the neural level, prior research has identified a diverse set of brain regions to play a role in decision-making and uncertainty. For example, confidence signals have frequently been observed in the medial prefrontal cortex (mPFC) (Bach and Dolan 2012; De Martino et al. 2013; Lebreton et al. 2015; Gherman and Philiastides 2018; Rouault et al. 2023), the anterior cingulate cortex (ACC) (Fleming et al. 2012; Bang and Fleming 2018) and the ventral striatum (Hebart et al. 2016; Bang and Fleming 2018), while the dorsolateral prefrontal cortex (dlPFC) has been implicated in outcome uncertainty and metacognition about uncertainty (Fleming et al. 2012; De Martino et al. 2013; Alexander et al. 2023; Rouault et al. 2023). Several of these regions are also involved in assessing the value and reliability of information originating from others, including the striatal and mPFC network, in connection with the ACC (Burke et al. 2010; Jones et al. 2011; Cooper et al. 2012; Suzuki et al. 2012; van den Bos et al. 2013; Morelli et al. 2015; Zaki et al. 2016). Areas that have been found to be specifically involved in social cognition and reasoning about others’ beliefs include the temporo-parietal junction (TPJ) (Saxe 2006; McKell Carter et al. 2012; van den Bos et al. 2013; Zaki et al. 2016) and mPFC (Amodio and Frith 2006; Hampton et al. 2008; Van Overwalle 2011; Apps and Ramnani 2017). For instance, learning about others’ expertise has been shown to be reflected in both the TPJ and mPFC (Boorman et al. 2013). Although these findings suggest that confidence signals about directly experienced information and social information are processed in partly overlapping brain areas, it is not yet clear how these two sources are combined to form a final decision. A comparison of self- and other-related processing suggests that social information processing, including mentalizing and confidence in others’ decisions, is associated with the more dorsal and ventral regions of the mPFC. In contrast, self-related processing, such as self-knowledge and uncertainty about personal decisions, is reflected in more anterior regions (Lockwood and Wittmann 2018; Lieberman et al. 2019; Jiang et al. 2022). Moreover, correlates of the integration of information have been observed in the ventromedial prefrontal cortex (vmPFC) (Behrens et al. 2008; Benoit et al. 2014; Zhang and Gläscher 2020). However, there is limited information from previous studies on how personal and social information and particularly the reliability associated with them are integrated at both the cognitive and neural levels.

Here, we employ two studies to investigate the cognitive processes related to social information use under uncertainty at the behavioral (experiments 1 and 2) and neural level (experiment 2). In study 1, participants perform a social information use task to examine the relative contribution of own versus social information at the behavioral level. In this task, participants have the opportunity to update an initial response based on a peer’s response. We vary both the amount of their personal information (own certainty) and the peer’s confidence rating (peer confidence). We hypothesize that participants use more social information when they themselves find themselves in an uncertain situation and when the other reports to be more confident, in line with a Bayesian framework. We therefore compare different Bayesian models to define the cognitive processes in terms of a precision modulation when participants combine their own information with social information. In line with earlier studies that found a systematic reliance on simple decision heuristics (Molleman et al. 2020), we also take into consideration that participants exhibit a bias to stay with their own initial decision, which does not integrate the peer’s response and is therefore separate from the Bayesian process. In study 2, we extend our procedure to an fMRI paradigm to explore which brain areas are involved in these decisions and how they are related to Bayesian processes. We particularly focus on regions implicated in uncertainty and confidence signals, including the mPFC, dlPFC, insula, cingulate cortex, and striatum, as well as on regions implicated in social cognition, including the mPFC and TPJ. Note that throughout the paper, we refer to a participant’s own certainty in terms of “certainty” and to the peer’s certainty in terms of “confidence,” purely to distinguish the two task manipulations; this choice is not intended to imply any underlying psychological difference between the two constructs, even though such a difference may exist.

## Material and methods

### Participants

#### Experiment 1

One hundred thirty-seven participants between 18 and 45 years old were recruited through Prolific (www.prolific.com) for the online behavioral experiment, of which 135 completed the task (30 min). They were paid 3.75 pounds plus a bonus contingent on their performance, ranging between 0 and 1.50 pounds. Thirty-four participants were excluded from data analysis (see below), resulting in 101 participants [50 women; age: mean (SD) = 24.0 (5.7), range 18 to 45].

#### Experiment 2

One hundred thirty participants were recruited via the research participant system of the University of Amsterdam and Instagram for the fMRI experiment. Participants were between 22 and 45 years old, right-handed, Dutch-speaking, screened for magnetic resonance compatibility, had completed or were following a university-level education and had normal or corrected-to-normal vision. The experiment described in the current study was part of a larger task battery, consisting of a session from home (30 min) during which participants filled in a questionnaire about their digital device and social media use and a behavioral-fMRI session at the research center (2.5 h) during which they completed a social information use task in the MR scanner (described below) as well as a spatial working memory task, a reversal learning task, an information search task, and a shorter social information use task (not reported here). Participants were paid 40 euro plus a bonus contingent on their performance, ranging between 0.70 and 2.90 euro for all tasks combined and ranging between 0 and 1.50 for the fMRI task. Out of the 131 participants who signed up for the study, a total of 74 completed their fMRI session. Two of those were excluded due to incomplete datasets, one was excluded because they fell outside our predefined age range, and a further 3 participants were excluded based on our exclusion criteria described below, resulting in 68 participants [40 women; age: mean (SD) = 24.8 (3.8), range 22 to 43]. Ten further datasets were excluded from the fMRI analysis because they did not pass our quality assessment, resulting in 58 participants [35 women; age: mean (SD) = 24.3 (3.5), range 22 to 43].

The procedure of both studies was approved by the local ethics committee (experiment 1: 2021-DP-13538; experiment 2: 2021-DP-13756). Before the start of the experiment, participants gave written consent according to the Declaration of Helsinki.

### Social information use task

#### Experiment 1

The social information use task is an estimation game modeled after the marble task (Phillips and Edwards 1966) and the Berlin Estimate AdjuStment Task (BEAST; Molleman et al. 2019) and probed the amount of social information use as a function of own prior certainty and others decision confidence (Fig. 1B). The game was programmed in JavaScript using PsychoPy version 2021 January 4 (Peirce et al. 2019) and hosted on Pavlovia (www.pavlovia.org).

Participants were instructed that they are part of a community for which the main food resource is mushrooms. These mushrooms are harvested from a forest in the vicinity of their village. Soon, it will be time to harvest, and the leader of the community has asked them for their help in coordinating this. There are two types of mushrooms, blue and red ones. Their task at the start of each day (trial) is to estimate the percentage of blue mushrooms in the forest. To make an informed guess, they will walk through 5 fields that surround their village. The mushrooms in the fields are a good but not exact representation of what will be found in the forest on that day. On some days, they encounter many mushrooms (45) in the fields, and, on other days, they encounter fewer mushrooms (5). The more mushrooms they find in the nearby fields, the more information they have to base their estimate on and the more certain they can be. After they have submitted their first estimate by typing it into a textbox, they will be shown peer information. The percentage of blue and red mushrooms in the forest was the same for this other person, but they were from a different village on the other side of the forest, meaning that they had to sample from different fields on the other side of the forest, and the number of blue and red mushrooms in these fields might have been different from those in the fields surrounding the participants’ village. The participant was first shown the peer’s confidence rating in their estimate, which could be low, medium, or high. After 2 s, this information was accompanied by the peer’s estimate. Based on this new information, the participant can adapt their estimate. The peers’ estimate and confidence rating stemmed from real previous participants, who had played a version of the mushroom game without any social information. The peer estimate was chosen such that, if possible, it was in the direction of the true underlying percentage and the difference between the participants' first estimate and the peer estimate ranged between 15 and 18 percentage points. This range was selected to leave enough room for adjustment of the estimate and control for distance that might influence the amount of adjustment (Molleman et al. 2020).

Participants completed 42 trials equally divided across own certainty (certain, uncertain), peer confidence (low, medium, high) and true percentage of blue versus red mushrooms (12.5, 25, 37.5, 50, 62.5, 75, 87.5). Another set of 12 “filler trials” was included to make the trials more realistic: The true percentage could range from 0 to 100, and *P* was either very close (<3) or very far apart (>40) from E1. These 54 trials in total were randomly divided across 3 rounds, with the possibility of having a break of maximum 2 min in between the rounds. The maximum time to submit an estimate was 10 s, after which the trial would time out and the participant would see a message stating that they were too late. The main task was preceded by instructions and 2 rounds of 3 practice trials. No feedback was given on their estimates, but participants could earn a bonus depending on their performance. One estimate of one trial was randomly selected. If this estimate was exactly the same as the true percentage, they would earn 1.50 pounds, of which 0.04 pounds was subtracted for each percentage point off. Their bonus could never go below 0.

#### Experiment 2

The task used for the fMRI experiment was similar to that in experiment 1, with only a few alterations to render it suitable for offline testing and probing neural activity. The game was programmed in Python 3.6.6 using PsychoPy. Rather than typing in their estimate, participants used an MR-compatible button box to select their estimate on a slider, for which there was a maximum response time of 20 s. The jittered intertrial interval was increased from 1.5 to 2 to 4 to 6 s, and the time from seeing the peer information to when they could submit their E2 was increased from 2 to 4 s to decrease overlap of hemodynamic responses between events. Crucially, both the peer’s confidence and their estimate were presented simultaneously, ensuring that any neural activity observed during the peer confidence phase reflected the participant’s full awareness of the peer’s estimate, rather than just their confidence in isolation without knowing the estimate itself. The total number of trials was 75, which were pseudo-randomly divided across 3 runs, which lasted approximately 30 min in total. Sixty trials were again equally divided across own certainty (uncertain, certain), peer confidence (low, medium, high), and true percentage of blue versus red mushrooms (12.5, 25, 37.5, 62.5, 75), and the other 15 trials were filler trials. Again, participants could earn a maximum of 1.50 euro based on the accuracy of a randomly selected estimate. The main task was preceded by instructions, 2 short rounds of 3 practice trials each, and a longer practice round of 15 trials outside the scanner. During this practice round, participants were asked to rate their own confidence after submitting each E1 as low, medium, or high, to give them a better understanding of what the peers’ confidence ratings entailed. Moreover, a quick analysis of this practice round informed the experimenter about whether the participant indeed understood the game before going into the scanner.

### MRI acquisition and preprocessing

#### Experiment 2

The fMRI experiment was performed on a Philips Achieva 3T MRI scanner and a 32-channel SENSE headcoil at the imaging center at the Roeterseiland campus of the University of Amsterdam. First, a 6-min whole-brain high-resolution structural image was acquired for anatomical referencing, using a T1-weighted magnetization prepared, rapid-acquisition gradient echo (MPRAGE) sequence (TR = 8.2 ms, TE = 3.7 ms, flip angle = 8°, 220 axial slices in ascending order, field of view = 240 × 188 mm, voxel size = 1 × 1 × 1 mm). Images with blood-oxygen level–dependent (BOLD) contrast were acquired in 3 runs, using a whole-brain T2*-weighted single-shot gradient-echo multi-echo echo planar imaging (EPI) sequence (TR = 2,000 ms, TE = 28 ms, flip angle = 76.1°, 36 axial slices per volume in ascending order, field of view = 240 × 240, reconstruction matrix = 80 × 80, voxel size = 3 × 3 × 3 mm, slice gap = 0.3 mm). After each functional run, a phase-encoding polarity (“topup”) gradient-echo sequence consisting of 3 volumes was acquired to estimate the static magnetic field’s inhomogeneity. The parameters were exactly the same as for the corresponding functional scans, but with a reversed phase-encoding direction.

All images were converted from PAR/REC to NIfTI format and into BIDS (Brain Imaging Data Structure) (Gorgolewski et al. 2016) using BIDScoin (Zwiers et al. 2022). The images were preprocessed using HALFpipe (Harmonized AnaLysis of Functional MRI pipeline) version 1.2.2 (Waller et al. 2022), which is based on fMRIPrep (Esteban et al. 2019). Structural images were corrected for bias field, skullstripped, and segmented before being registered to the MNI152NLin2009cAsym standard template. Preprocessing of the functional images included realignment to the middle volume, slice timing correction, susceptibility distortion correction based on the topup scans, coregistration to the participant’s structural scan, and spatial normalization to MNI152NLin2009cAsym space. Denoising was then performed by spatial smoothing using a 6 mm FWHM kernel, grand mean intensity normalization with a mean of 10,000, and temporal filtering using a high-pass filter width of 100 s. Physiological nuisance regressors were extracted using aCompCor (Behzadi et al. 2007). See https://fmriprep.readthedocs.io/en/0.6.2/workflows.html for more details on the preprocessing pipeline. Ten participants were excluded after data quality assessment, including a visual check of the skull stripping, spatial normalization, EPI tSNR, EPI confound, and head motion results (excessive motion was defined as mean FD > 0.5 mm or max FD > 3 mm).

### Behavioral data analysis

Our main outcome measure was social information use (*s*), defined as the adjustment from the first (*E*_1_) to the second (*E*_2_) estimate, relative to the peer estimate (*P*):


(1) \begin{equation*} s=\frac{E_2-{E}_1}{P-{E}_1} \end{equation*}

Social information use could therefore range from 0 (no social information use) to 1 (copying of *P*).

Participants who did not understand the task (exp. 1: *n* = 19; exp. 2: *n* = 1), as assessed per qualitative visual inspection, were excluded from the analysis (eg participants who responded randomly to differences in percentage of blue versus red mushrooms or participants who did not extrapolate the number of seen mushrooms to a total of 100), as were participants whose social information use was 0 at more than 70% of the trials (exp. 1: *n* = 15; exp. 2: *n* = 2), as this would yield minimal variation to conduct the analyses. Filler trials, missed trials (without a response at either the first or second estimation phase), trials in which no suitable social information (ie within the correct range from the participant’s first estimate) could be sampled from our database, trials for which either the first or the second estimate deviated more than 3 SD from the group mean, grouped per certainty condition and ratio, and trials in which social information use did not range between 0 and 1, meaning that it was not a weighted average of the participant’s first estimate and the peer’s estimate, were excluded from the analysis.

The behavioral analyses were conducted in R version 4.0.4 (R Core Team 2021). To assess the combined effects of own certainty and peer confidence on social information use, we conducted a linear mixed effects regression, using the lmer function from the *lmerTest* package (Kuznetsova et al. 2017). Own certainty and peer confidence (with mean-centered values −1 for low, 0 for medium, and 1 for high peer confidence), as well as their interaction, were included as both fixed and random effects.

### Computational modeling

We used a computational modeling approach to quantify Bayesian processes that we hypothesized to underlie participants’ choices (Fig. 1A). Apart from a base model M0, we developed nine models with increasing complexity to investigate whether additional parameters explained the observed behavior better. The model-building process was conditional: If a model performed worse compared to the preceding model (based on the Bayesian Information Criterion [BIC]; see Model Fitting Procedure below), we did not use it as the basis for further model development. Instead, the next model in the sequence was built upon the last accepted model. This iterative approach ensured that each new model only added complexity if it improved the model fit. An overview of all models can be found in Table 1. For all models, we assumed a beta distribution over all possible response options for their E2 (0% to 100% divided by 100). We set an initial prior of 1 blue and 1 red mushroom, to which we then added the participant’s estimate of blue and red mushrooms to arrive at a posterior for *E*1, where we further assumed that participants’ *E*1 was an accurate representation of the percentage of blue mushrooms they had seen in the fields.


(2) \begin{equation*} {\displaystyle \begin{array}{c}E{1}_{blue}=1+\frac{E1}{100}\times N\\{}\ E{1}_{red}=1+\left(1-\frac{E1}{100}\right)\times N\end{array}} \end{equation*}

where $N$ is the total number of mushrooms that the participant saw on that trial, being either 5 for the uncertain condition and 45 for the certain condition. For example, if 45 mushrooms were shown in a particular trial and the *E*1 was 20, meaning that the participant estimated that the percentage of blue mushrooms was 20%, the number of blue mushrooms was set to 20/100 × 45, which was then added to the prior of 1, resulting in $E{1}_{blue}=10$. The number of blue and red mushrooms as seen by the peer follow from the peer’s estimate *P* and the total number of mushrooms seen by the peer. Because the total number of mushrooms seen by the peer is unknown to the participant, we set this to 25 in **M0**, which is the average of the total number seen by the participant, across all trials.


(3) \begin{equation*} {\displaystyle \begin{array}{cc}{P}_{blue}=\frac{P}{100}\times{N}_{peer}& \\{}&, {N}_{peer}=25\\{}\ {P}_{red}=\left(1-\frac{P}{100}\right)\times{N}_{peer}& \end{array}} \end{equation*}

Adding these peer’s ${P}_{blue}$ and ${P}_{red}$ to the participant’s $E{1}_{blue}$ and $E{1}_{red}$, respectively, results in the posterior for E2:


(4) \begin{equation*} {\displaystyle \begin{array}{c}E{2}_{blue}=E{1}_{blue}+{P}_{blue}\\{}\ E{2}_{red}=E{1}_{red}+{P}_{red}\end{array}} \end{equation*}

One can then define a probability density function (PDF) of the posterior beta distribution based on ' $E{2}_{blue}$ and $ E{2}_{red} $, for $0\le x\le 1 $ and shape parameters $ E{1}_{blue}$ and $E{1}_{red}$:


(5) \begin{equation*}f\left(x;E{2}_{blue},E{2}_{red}\right)=\frac{x^{E{2}_{blue}-1}\times{\left(1-x\right)}^{E{2}_{red}-1}}{B\left(E{2}_{blue},E{2}_{red}\right)}\end{equation*}

where the beta function $B$ is a normalization constant to ensure that the total probability adds up to 1, which we then used for our model fitting procedure (see more details below).

**Table 1:** Computational models.

Model	Description	Parameters	ΔBIC	
			Experiment 1	Experiment 2
**M0**	Base model	ND	3199	4693
**M1a**	M0 + modulation of the weight of E1	$0.1\le \alpha \le 100$	2204	3644
**M1b**	M0 + modulation of the weight of E1, separately for the low and high own certainty condition	$0.1\le{\alpha}_{uncertain}\le 100$ $0.1\le{\alpha}_{certain}\le 100$	786	2940
**M2a**	M1b + modulation of the weight of the peer estimate	$0.1\le{\alpha}_{uncertain}\le 100$ $0.1\le{\alpha}_{certain}\le 100$ $0.1\le \theta \le 1000$	745	2152
**M2b**	M1b + modulation of the weight of the peer estimate, linearly dependent on peer confidence level	$0.1\le{\alpha}_{uncertain}\le 100$ $0.1\le{\alpha}_{certain}\le 100$ $0.1\le{\theta}_{IC}\le 500$ $0.1\le{\theta}_{slope}\le 500$	568	143
**M2c**	M1b + modulation of the weight of the peer estimate, separately for each peer confidence level	$0.1\le{\alpha}_{uncertain}\le 100$ $0.1\le{\alpha}_{certain}\le 100$ $0.1\le{\theta}_{low}\le 1000$ $0.1\le{\theta}_{medium}\le 1000$ $0.1\le{\theta}_{high}\le 1000$	1178	364
**M3a**	M2b + overall stay bias	$0.1\le{\alpha}_{uncertain}\le 100$ $0.1\le{\alpha}_{certain}\le 100$ $0.1\le{\theta}_{IC}\le 500$ $0.1\le{\theta}_{slope}\le 500$ $0.01\le \beta \le 0.99$	232	246
**M3b**	M2b + stay bias, separately for the low and high own certainty condition	$0.1\le{\alpha}_{uncertain}\le 100$ $0.1\le{\alpha}_{certain}\le 100$ $0.1\le{\theta}_{IC}\le 500$ $0.1\le{\theta}_{slope}\le 500$ $0.01\le{\beta}_{uncertain}\le 0.99$ $0.01\le{\beta}_{certain}\le 0.99$	799	632
**M3c**	M2b + stay bias, linearly dependent on peer confidence level	$0.1\le{\alpha}_{uncertain}\le 100$ $0.1\le{\alpha}_{certain}\le 100$ $0.1\le{\theta}_{IC}\le 500$ $0.1\le{\theta}_{slope}\le 500$ $0.01\le \beta \le 0.99$	58	55
**M3d**	M2b + stay bias, exponentially dependent on peer confidence level	$0.1\le{\alpha}_{uncertain}\le 100$ $0.1\le{\alpha}_{certain}\le 100$ $0.1\le{\theta}_{IC}\le 500$ $0.1\le{\theta}_{slope}\le 500$ $0.01\le \beta \le 0.99$	0	0

ΔBIC, Bayesian information criterion relative to the best-fitting model (lower BIC value indicates better fit); ND, no data; M3d (in bold) is the best-fitting model.

#### Model family 1: Individual modulation of the weight of E1

Participants might under- or overestimate the numerosity of displayed items or their own confidence (Lichtenstein and Fischhoff 1977; Krueger 1984). In **M1a**, we therefore tested whether adding a parameter $\alpha$ that modulates the perceived number of total mushrooms, effectively modulating the subjective weight of the *E*1 at the individual level, improved model performance:


(6) \begin{equation*} {N}_{perceived}=N\times \alpha \end{equation*}

In **M1b**, we extended this modulation by including a dual $\alpha$, which separately modulated the perceived number of total mushrooms for the uncertain and certain conditions:


(7) \begin{equation*} {N}_{perceived}=\left\{\begin{array}{c}N\times{\alpha}_{uncertain}\ if\ uncertain\ condition\ \\{}N\times{\alpha}_{certain}\ if\ certain\ condition\ \end{array}\right. \end{equation*}

#### Model family 2: Individual modulation of the weight of the peer estimate

Next, we tested whether individually modulating the number of mushrooms the participant assumed the peer had seen improved model performance. This accounts for the possibility that a participant selectively ignores the peer estimate. In **M2a**, rather than fixing the number of mushrooms as seen by the peer to 25, we introduced $\theta$ that modulated this number per participant:


(8) \begin{equation*} {N}_{peer}=\theta \end{equation*}

In **M2b**, we tested whether model performance improved when the modulation by $\theta$ linearly depended on the confidence level reported by the peer, such that the weight of the peer estimate increased as the peer reported to be more confident:


(9) \begin{equation*} {N}_{peer}={\theta}_{IC}+{\theta}_{slope}\times \left({confidence}_{peer}-1\right) \end{equation*}

where the confidence level was defined as 1 for low, 2 for medium, and 3 for high confidence. Here, when the intercept (IC) is estimated to be close to zero, participants effectively ignore the peer estimate in trials where the peer expresses low confidence. If both the IC and slope are estimated to be close to zero, no social information is integrated at any confidence level. In **M2c**, we tested whether a modulation by $\theta$ for each peer confidence level separately, allowing for a nonlinear modulation, improved model fit:


(10) \begin{equation*} {N}_{peer}=\left\{\!\!\!\!\!\ \begin{array}{c}{\theta}_{low}\ \\{}{\theta}_{medium}\\{}{\theta}_{high}\ \end{array}\right.{\displaystyle \begin{array}{c}\ if\ peer\ confidence\ is\ low\ \\{}\ if\ peer\ confidence\ is\ medium\\{}\ if\ peer\ confidence\ is\ high\ \end{array}} \end{equation*}

#### Model family 3: Stay bias

Following earlier findings suggesting that people combine Bayesian processing with simple heuristics, we added a stay bias to models M3. This bias allowed participants to exhibit a bias in staying with their first estimate while ignoring the peer’s estimate. For **M3a,** the stay bias, defined by parameter $\beta$, was nonselective, such that it was set per individual, but equal across all trials within-participant. **M3b** included a separate parameter β for each own certainty condition, ${\beta}_{uncertain}$ and ${\beta}_{certain}$, which allowed participants to effectively ignore the peer estimate when they were very certain themselves. For **M3c** and **M3d**, the stay bias depended on peer confidence level in a linear or exponential fashion, respectively. This allowed participants to selectively ignore the peer information under low peer confidence—when it was more likely to be unreliable:


(11) \begin{equation*} stay\ bias=\left\{\!\!\!\!\ \begin{array}{c}\beta\ \\{}{\beta}_5\ or\ {\beta}_{45}\ \\{}\beta /{confidence}_{peer}\\{}{\beta}^{confidence_{peer}}\ \end{array}\right.{\displaystyle \begin{array}{c}\ for\ M3a\\{}\ for\ M3b\\{}\ for\ M3c\\{}\ for\ M3d\end{array}} \end{equation*}

For these M3 models, the PDF was then multiplied by $\left(1- stay\ bias\right)$, such that the sum of the stay bias and the total area under the curve (the probability) of the PDF adds to 1. The probability of a stay response at *E*2 was then estimated by the sum of the stay bias and the probability of s = 0 under the PDF. This means that a stay response could be either the result of a simple stay bias or of a decision process that considered the peer’s estimate. On nonstay trials, the probability of participants’ responses was estimated solely by the probability of the E2 response under the PDF (multiplied by $1- stay\ bias$).

#### Model fitting procedure

Parameter estimations were performed by individually fitting the model predictions to each participant’s behavior, specifically their *E*2 responses. The fitting procedure relied on a combination of grid search and maximum likelihood estimation using the L-BFGS-B algorithm implemented in the optim function in R. The log likelihood of the combination of parameter values corresponding to each point on the grid was estimated and minimized. Model comparison was evaluated by computing the BIC, which penalizes for increasing model complexity, across all participants for each of the models. Lower BIC values indicate a better fit.

#### Model recovery and parameter recovery

For quality control, we ran a model recovery procedure using nine models (excluding base model M0). For each model, we simulated a dataset consisting of 169 simulated participants (101 corresponding to experiment 1 and 68 corresponding to experiment 2). To simulate realistic parameter values, we sampled from a uniform distribution bounded by the minimum and maximum values observed in the real data after excluding outliers beyond ±3 SD. Each of these simulated datasets was then fit by all nine models—resulting in 81 model fitting procedures. These fits were compared regarding their goodness-of-fit (BIC), and a confusion matrix was constructed to quantify model recoverability (Wilson and Collins 2019).

We further ran a parameter recovery procedure on all our models, where we assessed the correlation between the “true” parameter estimates, which we defined as the simulated parameter values, and the fitted parameter estimates, which were generated by rerunning each model on simulated data based on these “true” estimates in combination with the same settings and trials as in the experiment.

### fMRI data analysis

fMRI data processing was carried out using FEAT (FMRI Expert Analysis Tool) Version 6.00, part of FSL (FMRIB’s Software Library, www.fmrib.ox.ac.uk/fsl). Time-series statistical analysis was carried out using FMRIB's Improved Linear Model (FILM) with local autocorrelation correction (Woolrich et al. 2001). For each run of each subject, BOLD responses were modeled using event-related General Linear Models (GLMs). The first GLM included two parametric regressors: one that modeled model-based own certainty at the time of the *E*1 [ie the prior; first 500 ms, so brain activity would reflect (un)certainty rather than motor activity; Fig. 1B] and one that modeled model-based peer confidence at the time of the social information (duration of 4,000 ms). Model-based own certainty was the actual number of mushrooms that was shown on that trial multiplied by either ${\alpha}_{uncertain}$or ${\alpha}_{certain}$[see Eq. (7)], depending on the trial condition. Model-based peer confidence was defined as ${\theta}_{IC}+{\theta}_{slope}\times \left({confidence}_{peer}-1\right),$ with peer confidence set as low = 1, medium = 2, high = 3 [see Eq. (9)]. We reasoned that model-based own certainty and peer confidence, based on the parameter values resulting from our best-fitting Bayesian model, yielded more individualized results. However, we also ran a supplemental GLM that included two regressors relating to the model-free conditions of own certainty, modeled at the time of the *E*1 (first 500 ms), one for the certain and one for the uncertain condition, and one regressor relating to model-free peer confidence modeled at the time of the social information (duration of 4,000 ms). The latter was parametrically modulated by the peer confidence level. We created contrasts for the effect of own certainty (certain—uncertain at *E*1) and the effect of peer confidence (parametric effect at social information).

To model belief updating in response to peer information, we modeled another GLM that included a parametric, model-based regressor at the time of the social information (4,000 ms) that represented the Kullback–Leibler (KL) divergence between the participant’s first and second estimate. The KL divergence is a measure that expresses how much the beta distributions of *E*1 and *E*2 differ from each other, accounting for both the shift in the point estimate (equivalent to *s*), as well as the width of the distributions. For example, when a participant is relatively uncertain about their *E*1 and *E*2, their beta distributions are wide (Fig. 1A, upper panel), whereas when a participant is relatively certain, their distributions are narrow (Fig. 1A, lower panel). The same shift in estimate (or the same arrow length in Fig. 1A), would therefore reflect a larger underlying belief update where the distributions overlap less in the latter case compared to the former case. Thus, KL divergence reflects a belief update based on Bayesian principles that accounts for uncertainty surrounding a decision, with higher KL divergence reflecting a larger update. KL divergence was computed in R using the KL function from the *philentropy* package (Drost 2018).

Although not central to our research question, we supplementary tested for neural correlates of the stay bias. We ran an additional GLM in which stay bias, exponentially modulated by peer confidence, was included as a parametric modulator at the time of the social information (Table S6).

The parametric regressors were mean-centered across all trials within a run. Of note, peer confidence and KL divergence, which were modeled at the time of social information, only included nonfiller trials because filler trials contained peer information (very close or very far from the *E*1) that might have elicited different cognitive processes. In all models, we additionally included temporal derivatives, six motion parameters, framewise displacement, global white matter and cerebrospinal fluid (CSF) signal and the first 6 anatomical principal component noise regressors (aCompCor) as regressors of no interest. All regressors were convolved with a double-gamma hemodynamic response function.

The resulting contrast images per run were averaged at the participant level, which was carried out using a fixed effects model, by forcing the random effects variance to zero in FLAME (FMRIB’s Local Analysis of Mixed Effects) (Beckmann et al. 2003; Woolrich et al. 2004; Woolrich 2008). The participant-level contrasts were subsequently taken to the group level, where they were submitted to a one-sample *t*-test using FLAME stage 1. Normalized (*Z*-scored) statistic images were thresholded using clusters determined by *Z* > 3.1 and a (corrected) cluster significance threshold of *P* = 0.05 (Worsley 2001). For the group-level analyses, we applied a mask that included the frontal lobe and insula, derived using the Montreal Neurological Institute (MNI) structural atlas in FSLeyes, as well as the TPJ and the striatum, based on connectivity analyses (Mars et al. 2012; Piray et al. 2017) (Fig. 1C). To also give a complete overview, results at the level of the whole brain can be found in the supplemental material (Tables S3–S5).

Based on the results of our univariate fMRI analyses, we conducted an exploratory connectivity analysis, conceptually similar to a psychophysiological interaction (PPI) analysis, to examine whether functional coupling between mPFC regions is modulated by belief updating. We focused on one cluster in the mPFC that negatively correlated with perceived own certainty (thus, positively with perceived uncertainty), and three distinct clusters in the mPFC that positively correlated with perceived peer confidence. For each contrast, we created a group-level Region of Interest (ROI) mask (*Z* > 3.1, cluster-corrected) and then identified the peak voxel for each individual within this mask. Around each subject-specific peak, we defined a 3 mm spherical ROI and extracted the mean timeseries. We used the timeseries from the uncertainty-related ROI as the seed region and the timeseries from each of the three peer-confidence-related ROIs as separate target regions. For each seed–target pair, we ran a linear mixed-effects model at the trial level using the using the lmer function from the *lmerTest* package in R. The model included the target region’s timeseries as the dependent variable and the interaction between the seed region’s timeseries and belief updating (KL divergence) as the key predictor, along with the main effects of each predictor and by-subject random slopes and intercepts. This yielded three interaction models, one for each target region. The primary outcome of interest was the interaction term (the PPI effect), indicating whether belief updating modulated functional coupling between the regions.

In a second step, we extracted the subject-level random slopes for the seed region’s main effect on the target region (ie individual coupling strength) and correlated these values with each participant’s mean KL divergence across trials, to assess whether individual variability in connectivity was associated with individual differences in belief updating.

## Results

### Both uncertainty and peer confidence increase social information use

#### Experiment 1

**Fig. 2:**
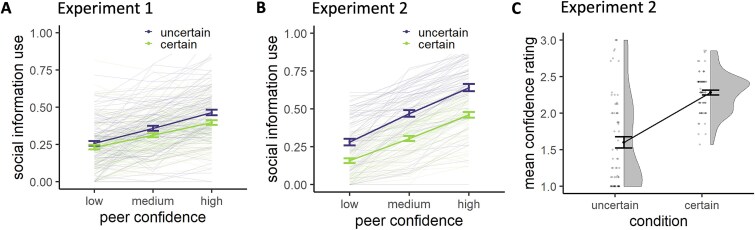
Effects of own certainty and peer confidence on social information use. A) Social information use per own certainty and peer confidence level in online “experiment 1.” Error bars represent the standard error around the mean. Thin lines represent individual participants. B) Social information use per own certainty and peer confidence level in fMRI “experiment 2.” Error bars represent the standard error around the mean. Thin lines represent individual participants. C) Mean self-reported confidence rating in experiment 2, averaged per own certainty condition, collected during the 15 practice trials.

The average social information use across all trials was 0.34, which is comparable to previous studies (Yaniv 2004; Molleman et al. 2019; Gradassi et al. 2022). Social information use was lower in the certain compared to the uncertain condition (*b* = −0.05, *P* < 0.001) and increased with increasing peer confidence (*b* = 0.09, *P* < 0.001). There was also an interaction between own certainty and peer confidence (*b* = −0.02, *P* = 0.012), such that the effect of peer confidence was stronger in the uncertain compared to the certain condition (Fig. 2A).

#### Experiment 2

An initial analysis on the participants’ mean confidence ratings, which they reported after each trial during the practice round in this experiment (1 = low, 2 = medium, 3 = high confidence), showed that participants were subjectively more confident in the certain condition compared to the uncertain condition [*t*(66) = 7.41, *P* < 0.001; Fig. 2C], thereby validating our task manipulation. The average use of social information across all trials was 0.39, which is slightly higher in this experiment compared to the online experiment but remains within a similar range. Again, social information use was lower in the certain compared to the uncertain condition (*b* = −0.16, *P* < 0.001) and increased with increasing peer confidence (*b* = 0.17, *P* < 0.001). There was again a significant interaction between own certainty and peer confidence (*b* = −0.03, *P* = 0.002), such that the effect of peer confidence was stronger in the uncertain compared to the certain condition (Fig. 2B). Thus, participants showed an egocentric bias, particularly when the peer reported low confidence, assigning roughly three times more weight to their own information compared to the peer’s. This bias tended to become more balanced when participants were uncertain and the peer expressed higher.

### Bayesian mechanisms of social information use

**Fig. 3:**
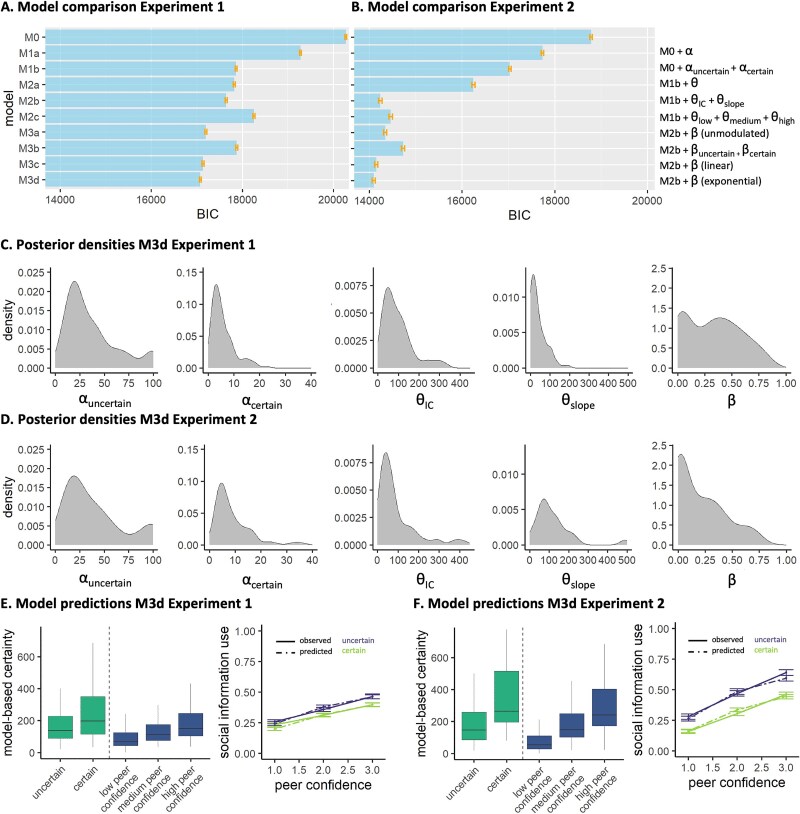
Model evidence and parameter inference. Model evidence, relative to the best-fitting model M3d, for “experiment 1” A) and “experiment 2” B). Stepwise addition of a parameter that modulates the weight of the personal information (α) a parameter that modulates the weight of the peer information (θ) and a stay bias (β) improves model fit, quantified by BIC. Lower BIC indicates better model fit. Posterior densities of the best-fitting model M3d for “experiment 1” C) and “experiment 2” D). Posterior predictive model simulations of the best-fitting model M3d for “experiment 1” E) and “experiment 2” F). The left panel of each figure shows model-based certainty for each of the own certainty levels (green), computed as the actual number of mushrooms seen by the participant multiplied by the respective α of each condition, and model-based certainty for each of the peer confidence levels (blue), computed as a linear combination of θ_IC_ and θ_slope_. The right panel of each figure shows the predicted (dotted lines) and observed (solid lines) for each own certainty and peer confidence level.

We used a computational modeling approach to quantify Bayesian mechanisms hypothesized to govern social information use. Specifically, we sought to disentangle how both own certainty and peer confidence contribute to social information use in a Bayesian fashion. Model comparison yielded that, for both experiments, the model that best captured the data was M3d (Fig. 3A, B, Table 1), which included a dual α to modulate the impact of own certainty on the weight of the personal information, a ${\theta}_{IC}$ and a ${\theta}_{slope}$ to linearly modulate the impact of peer confidence on the weight of the peer estimate, and a stay bias $\beta$ to capture participants' tendency to stay with their initial estimate, which became exponentially smaller as peer confidence increased. Figure 3C–F illustrates the parameter estimates and model predictions per experiment.

The improvement of model fit due to the dual α parameter indicates that participants differentially converted the actual number of mushrooms in each condition into a subjective count, thereby reflecting differential translation into model-based certainty. In “experiment 1,” participants amplified their model-based certainty more in the uncertain condition (median ${\alpha}_{uncertain}$ = 28) compared to the certain condition (median ${\alpha}_{certain}$ = 4). When we multiply participants’ ${\mathrm{\alpha}}_{uncertain}$ and ${\mathrm{\alpha}}_{certain}$ with the actual number of mushrooms, we see that their model-based certainty was significantly lower in the uncertain (median = 140) versus the certain condition (median = 180; *V* = 854, *P* < 0.001; Fig. 3E). In “experiment 2,” participants amplified their model-based certainty more in the uncertain condition (median ${\alpha}_{uncertain}$ = 29) compared to the certain condition (median ${\alpha}_{certain}$ = 6). When we multiply participants’ ${\mathrm{\alpha}}_{uncertain}$ and ${\mathrm{\alpha}}_{certain}$ with the actual number of mushrooms, we see that their model-based certainty was significantly lower in the uncertain (median = 145) versus the certain condition (median = 270; *V* = 193, *P* < 0.001; Fig. 3F). Thus, participants behaved “as if” they were almost 1.5 to 2 times as certain in the certain versus the uncertain condition, even though the actual ratio between 45 and 5 is 9. Participants thus discounted the condition difference, mainly driven by a relatively exaggerated model-based certainty in the uncertain condition.

Model performance further improved after the addition of the $\theta$ parameters, providing clear evidence of participants behaving “as if” the peer had seen more mushrooms for higher reported peer confidence. For “experiment 1,” participants behaved “as if” the peer had seen 72 mushrooms for low confidence, 115 mushrooms for median confidence, and 150 mushrooms for high confidence (as per Eq. (9); median values; Fig. 3E), thereby leading to a stronger weighting of the peer’s estimate in the participant’s second estimate if the peer reported to be more confident. For “experiment 2,” participants behaved *as if* the peer had seen 55 mushrooms for low confidence, 149 mushrooms for median confidence, and 241 mushrooms for high confidence (as per Eq. (9); median values; Fig. 3F).

Finally, model performance improved by adding a stay bias that depended on the peer confidence level but not on own certainty, showing that peer characteristics are the primary driver of this decision heuristic. These observations suggest that high peer confidence led participants to decide to use social information at all, after which both own certainty and peer confidence influenced the extent to which they relied on the social information.

#### Model recovery and parameter recovery

The model recovery results show that data generated by one specific model generally also resulted in the best model fit for that same model, indicating successful model recovery (Fig. S1). Importantly, simulations were not falsely fit best by our winning model M3d, suggesting that M3d does not overfit data generated by other models. However, there was some overlap in model fits between M3c and M3d, which is unsurprising given their strong similarity (a stay bias that depends linearly versus exponentially on peer confidence level).

Our parameter recovery procedure on the winning model M3d revealed high Pearson’s correlations between the “true” and fitted parameter estimates (all *r* ≥ 0.62, all *P* < 0.001; Table S1), suggesting that the model was adequately able to recover the parameters (see Table S2 for correlations between the different parameters).

### Neural correlates of own certainty, peer confidence, and belief updating

**Fig. 4:**
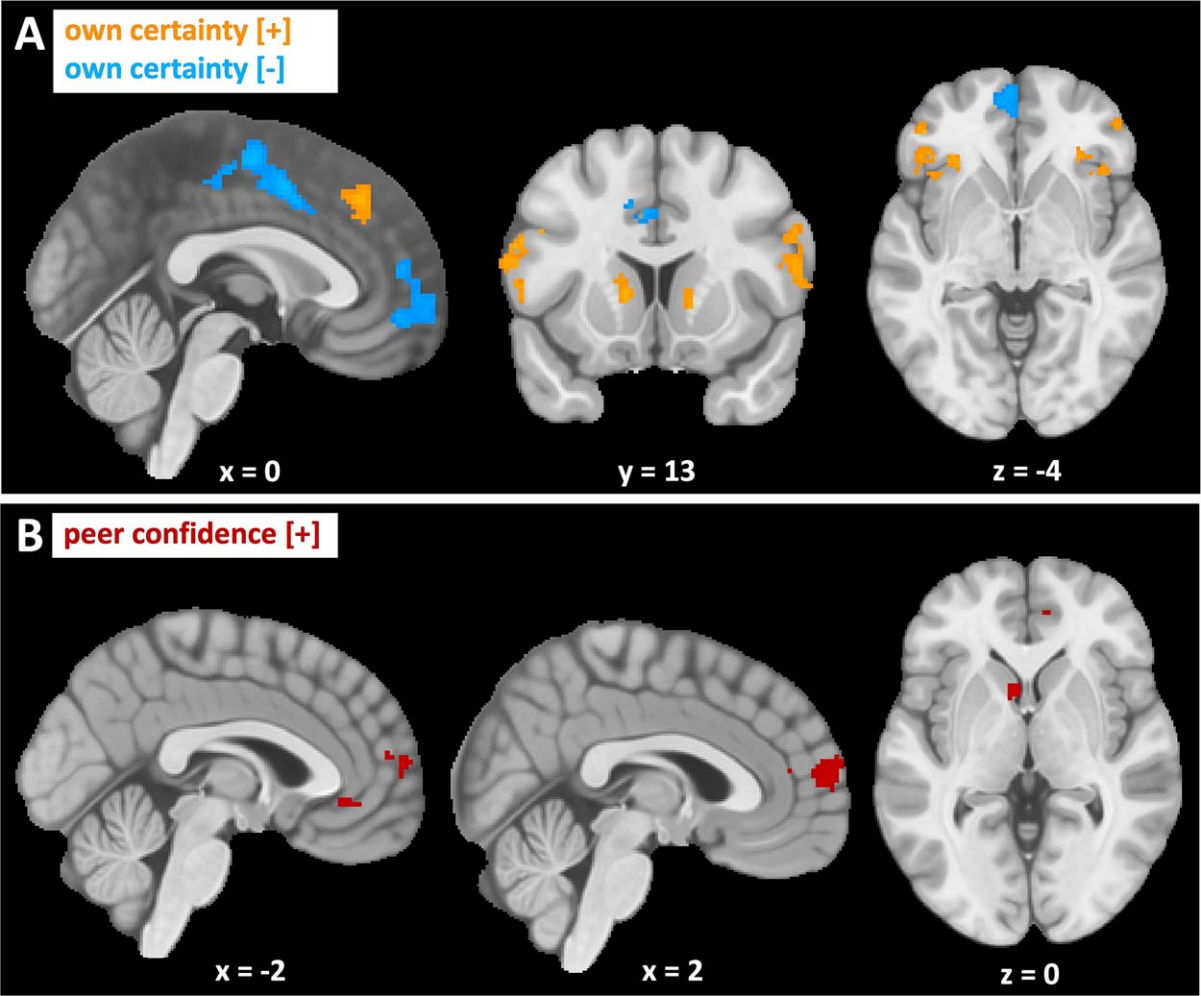
Neural activity in response to model-based own certainty and peer confidence. Clusters showing BOLD signal that A) significantly increased (orange) or decreased (blue) in response to own certainty and B) significantly increased (red) in response to peer confidence. Cluster-level corrected, FWE, *P* < 0.05. Coordinates correspond to MNI space.

We first assessed neural activity at the time of the first estimate as a function of model-based own certainty (Fig. 4A, Table S3). There was a large network of regions that correlated positively with own certainty (certain > uncertain), including the bilateral dorsolateral PFC, the (dorso)medial PFC, the bilateral anterior insula, and the left caudate nucleus. Regions that correlated negatively with own certainty (uncertain > certain) were the left premotor gyrus, the left midcingulate gyrus, and the medial PFC. Results from our supplemental GLM, which included model-free regressors of own certainty and peer confidence, produced similar findings to those obtained using individually modeled regressors, supporting the neural basis of our computational model (Table S3; Fig. S2C). When examining neural activity at the time of the social information as a function of model-based peer confidence, we identified different regions that showed a positive correlation, including the medial PFC, the ACC, and the caudate nucleus (Fig. 4B, Table S3). There were no clusters that correlated negatively with peer confidence. Again, we found similar clusters for model-free peer confidence (Table S4; Fig. S2D).

**Fig. 5:**
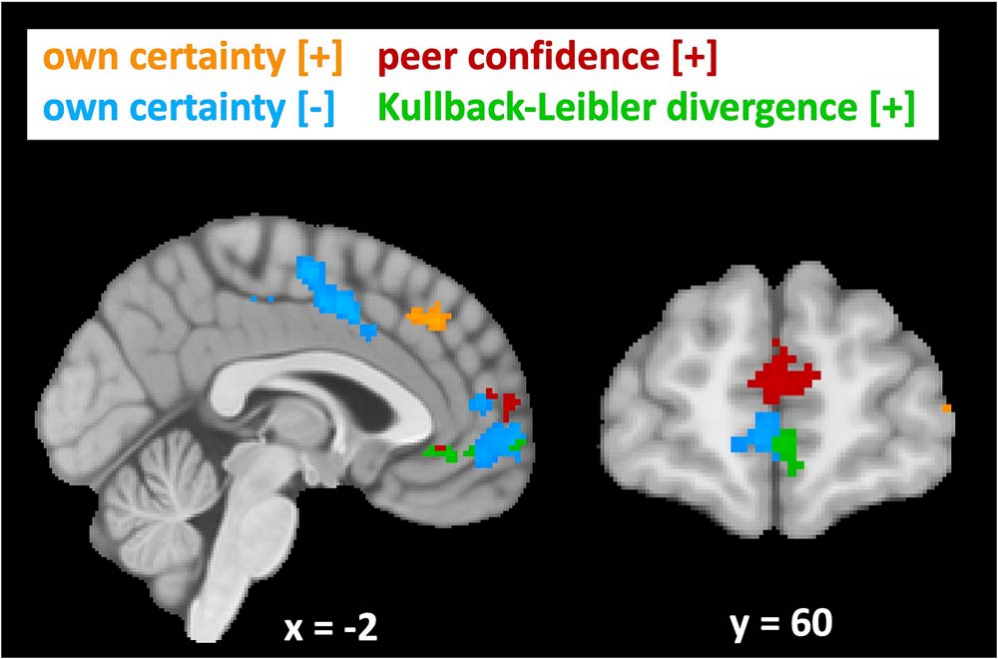
Neural activity in response to model-based own certainty, peer confidence, and Kullback–Leibler (KL) divergence. Neural activity in the ventral and anterior regions of the medial PFC reveals a partial overlap between being uncertain about one’s own estimate (blue), being more confident about the peer’s estimate (red) and one’s belief update as defined by KL divergence (green). Own certainty and peer confidence were included in the same GLM, whereas KL divergence was modeled using a separate GLM. Cluster-level corrected, FWE, *P* < 0.05. Coordinates correspond to MNI space.

We then analyzed neural activity in response to participants’ belief update, defined by KL divergence, a latent variable extracted from our Bayesian model that takes into account the (im)precision surrounding participants’ beliefs. We found positively correlating clusters in the ACC and medial PFC (Table S5). Figure 5 shows the overlap in the medial frontal areas between activity in response to own certainty, peer confidence, and KL divergence. Activity related to peer confidence and KL divergence appeared to overlap, especially in the ACC. Since KL divergence is inherently correlated with our model-based measures of own certainty and peer confidence—where higher KL divergence corresponds to higher certainty or precision of either one’s own or the peer’s estimate—we conducted a post hoc analysis. This analysis included both own certainty and peer confidence, as well as KL divergence. This way, the results exclude any shared variance between the regressors, with only the variance that is uniquely explained by any of the regressors remaining. This revealed that peer confidence, but not KL divergence, still explained variance in medial PFC and ACC (Fig. S3).

### Exploratory connectivity analysis

Motivated by the observation that activity related to both own certainty and peer confidence partially overlapped in the medial PFC, we performed an exploratory functional connectivity analysis to examine whether belief updating modulated coupling between these regions. Specifically, we extracted timeseries from individual ROIs based on group-level clusters that responded to their own uncertainty (one ROI) and peer confidence (three ROIs) and used linear mixed-effects models to estimate whether trial-by-trial KL divergence modulated the functional relationship between these regions. As expected, activity in the uncertainty-related ROI temporally correlated with activity in the peer-confidence-related ROIs (all *t* ≥ 3.50, all *P* < 0.001). However, these analyses did not reveal any statistically significant PPI effects for the interaction between KL divergence and functional coupling between the uncertainty-related seed ROI and any of the three peer-confidence-related target ROIs (all |*t*| ≤ 0.753, all *P* ≥ 0.455). Furthermore, individual differences in connectivity strength (random slope of the seed region on the target region) were not significantly correlated with mean KL divergence across participants (all *r* ≤ 0.16, all *P* ≥ 0.241).

## Discussion

### Bayesian information-processing and a stay heuristic together predict social information use

In this study, we examined the neurocomputational constituents of social information use under own uncertainty and peer confidence. Overall, participants tended to rely more on their personal information than on social information, consistent with previous findings that identified this pattern of egocentric discounting. Notably, experiments 1 and 2 both showed that social information use increased when participants were more uncertain and when the peer reported to be more confident, where the combined effect could even override egocentric discounting, leading participants to rely more on social information than on their personal information. These central findings corroborate previous research where participants were more likely to use social information or search for more information when they find themselves in an uncertain situation (Kendal et al. 2004, 2018; Toelch et al. 2014; Desender et al. 2018; Olsen et al. 2019; Friedemann et al. 2023) and when the social source is deemed more trustworthy (Zarnoth and Sniezek 1997; Sniezek and Van Swol 2001; Olsen et al. 2019). These strategies, including that of “copy-when-uncertain,” are widely shared among different animal species, from bumblebees to rats (Laland 2004; Galef et al. 2008; Smolla et al. 2016; Jones et al. 2019), suggesting a general utility of these processes for evolutionary purposes. Our finding that the effect size of the peer’s confidence was larger than that of own certainty is in line with a recent study that showed a relatively larger impact of the confidence of others compared with participants’ own confidence in predictions about the US elections and in a perceptual judgment task similar to the currently used social information use task (Gradassi et al. 2022). Interestingly, we show that reported confidence statements aligned strongly with own certainty, as manipulated within the task. This suggests that peer confidence reports can serve as reliable indicators of the trustworthiness of social information and how much weight it should be given, even in the absence of objective precision estimates.

A computational model based on Bayesian information processing was able to capture participants’ behavior, aligning with the model-free results while providing deeper insights by deconstructing the cognitive process into distinct components. Participants’ behavior was best predicted by precision weighting of their own first estimate and that of the peer, combined with a separate bias to stay with their own first estimate, which was informed by peer confidence. This means that people assigned a higher weight to their personal information in the certain compared to the uncertain condition, and they assigned a higher weight to social information if the peer reported to be more confident, as this information was perceived to be more trustworthy or precise. Indeed, many decision-making processes have also been shown to be of a Bayesian nature, including visual perception, sensory-motor learning, and reversal-learning (Konrad and Daniel 2004; Stocker and Simoncelli 2006; Jang et al. 2015; van Bergen et al. 2015) and more recently social information use under risk and uncertainty (De Martino et al. 2017; Ciranka and van den Bos 2020). Related work has also explored how Bayesian social learning is altered in clinical groups such as individuals with borderline personality disorder and high autistic trait scores (Henco et al. 2020; Sevgi et al. 2020). These studies emphasize the broad relevance of Bayesian approaches to social learning, while the present work focuses on healthy individuals and neural mechanisms of integrating precision estimates. Moreover, in keeping with recent findings using a similar task, which showed how social information use could be captured by a model that combined Bayesian updating and a stay heuristic that depended on the distance of the social information to the participant’s prior (Molleman et al. 2020), our model most accurately described the data when we combined the Bayesian process with a simple heuristic that defined a stay bias that depended on the peer confidence rating but not participants’ own uncertainty. Together, these findings suggest that people integrate social information in a manner consistent with Bayesian principles—updating more when they are uncertain—although low-quality information is often ignored, even though it could, in principle, improve estimates when sufficiently taking into account the lack of precision.

### Medial prefrontal activity is related to own certainty, peer confidence, and belief updating

Next, we analyzed neural activity in relation to both own certainty and peer confidence to gain deeper insights into the neural mechanisms underlying these processes. This revealed that neural activity correlated positively with own certainty in the dorsolateral and dorsomedial PFC, the anterior insula, and the caudate nucleus, whereas a negative correlation was found mainly in the premotor area and mPFC. Peer confidence elicited stronger BOLD activity mainly in the caudate nucleus, the mPFC, and the ACC. The involvement of many of these areas might elicit little surprise, given their established roles in decision-making. For example, the striatum, including the caudate nucleus, has long been implicated in value-orienting behavior (Delgado et al. 2004; Grahn et al. 2008) and is therefore highly likely to signal the higher value of more precise information. The dlPFC and anterior insula have been linked to processing risk and uncertainty (Singer et al. 2009; Badre et al. 2012; Feldmanhall et al. 2019). The activation of the anterior mPFC aligns with prior studies that have demonstrated involvement of this area, together with the nearby orbitofrontal cortex, in subjective decision confidence in rodents (Kepecs et al. 2008; Masset et al. 2020) as well as primates, including humans (De Martino et al. 2013; Lebreton et al. 2015; Gherman and Philiastides 2018; Rouault et al. 2023). The ventral parts of the mPFC have also been implicated in social cognition (Amodio and Frith 2006; Hampton et al. 2008; Lockwood and Wittmann 2018), possibly explaining our current findings pertaining to peer confidence. Our key novel finding, however, is that by varying both personal certainty and peer confidence within the same task, we observed increased neural activity in nearby and partially overlapping regions of the ventral mPFC, both when participants were more uncertain and when the peer expressed greater confidence. Additionally, internal belief updating, computed as KL divergence, correlated with increased BOLD signal in the ventral mPFC and ACC, overlapping with regions associated with peer confidence. This suggests that this area is likely a hub for integrating information to update beliefs. This resonates with earlier studies. For example, a study in which participants played a dot-motion task revealed that the human perigenual ACC tracked decision confidence but not its component parts of sensory reliability or motion coherence and choice distance, which were cleverly decoupled from each other, suggesting a key role for this area in the integration of reliability information (Bang and Fleming 2018). More akin to the current design are observations that the ventral mPFC tracked the value of specific choices from both first-hand experiences and other people’s choices (Zhang and Gläscher 2020), which was additionally modulated by the confidence that was associated with other people’s choices (Campbell-Meiklejohn et al. 2017). Another study showed that when participants had to provide judgments about consumer goods both before and after viewing online reviews, the change in rating followed Bayesian integration principles, with a stronger update for low initial certainty and high reliability of the reviews. The ventral mPFC tracked both the final liking rates and the final certainty of these ratings, whereas the magnitude of the Bayesian update, computed as KL divergence, correlated with neural activity in the more dorsal mPFC (De Martino et al. 2017).

Our results show that uncertainty-related activity in the ventral mPFC was already present at the time of the first estimate, before any information about the peer had been displayed. This strongly suggests that this area is involved not only in the integration of different sources of information itself but also already in the preparation of the integration process. Activity in this area before the onset of the peer information might have been an anticipatory signal to prepare the brain for potentially valuable new information that should be integrated into a belief update. This interpretation dovetails recent accounts that reconcile a role for the ventral mPFC in both value updating and confidence representations (Lebreton et al. 2015; De Martino et al. 2017; Shapiro and Grafton 2020; Hoven et al. 2022). While the ventral mPFC has traditionally been implicated in the computation of value, these accounts suggest that this region is additionally involved in probabilistic representations of values. Confidence around value representations may automatically emerge to guide decision-making by signaling the reliability and therefore utility of new outcomes or information. Thus, value computation and Bayesian belief updating are not mutually exclusive processes. In our task, participants needed to evaluate the reliability of both their own and social information to decide how much social information should be incorporated. This view also helps explain why we found a negative correlation between ventral mPFC activity and own certainty, which might seem at odds with earlier studies reporting a positive correlation with certainty or confidence (De Martino et al. 2013, 2017; Lebreton et al. 2015; Hoven et al. 2022). We believe that this might be due to a different task structure. In previous work, participants’ certainty rating was tied to their final decision on each trial. In contrast, in our study, own certainty was an intermediate step within the decision-making process, guiding participants in deciding how much social information to integrate in their final response. Thus, ventral mPFC activity may reflect ongoing belief updating rather than static confidence in an already-committed choice.

Additional insight into the ventral mPFC’s role in integrating self and other information comes from earlier work by Mitchell and colleagues (Mitchell et al. 2006), who found overlapping activation in the ventral mPFC when making judgments about oneself and about a similar other, whereas mentalizing about a dissimilar other engaged more dorsal areas. Relatedly, Wittmann and colleagues (Wittmann et al. 2016) found that activity in the perigenual ACC reflected participants’ own recent performance and predicted self-evaluations, while dorsal mPFC activity tracked the degree to which others’ performance influenced self-evaluations—indicating a self–other integration. In our task, participants had no distinctive information about the peer, which may have led them to treat the peer as similar to themselves. This might account for the overlapping but not identical activation patterns for own uncertainty and peer confidence in the ventral mPFC, indicating partly separate processes that still allow for integration when the self–other boundary is small.

While we found clear functional coupling between subregions of the ventral mPFC that were associated with own uncertainty and peer confidence, respectively, this coupling was not modulated by KL divergence, and individual differences in connectivity strength did not predict belief updates. One reason for these null findings could be that our study design (eg trial number, sample size) was suboptimal to detect subtle trial-by-trial changes in connectivity. Alternatively, the integration of social information may not depend on modulation of connectivity but instead depend on stable, functionally coupled regions that each represent different inputs to the belief update. In this case, integration would occur at the level of representational content, with the ventral mPFC acting as a convergence zone. Future work using for example representational similarity analyses could prove useful to distinguish between these possibilities.

While our findings provide valuable insights, several limitations should be taken into account. One limitation is that our current design makes it challenging to definitively tease apart the separate neural correlates of peer confidence and belief updating, as participants most likely start the integration of the personal and peer information immediately upon seeing the peer information. To ameliorate this, one could reverse the order of the first-hand experience and the peer information in half of the trials, such that both pieces of information are isolated at the first timepoint. However, it is important to note that KL divergence will, by definition, remain correlated with both peer confidence and own certainty.

Another limitation lies in the way information was presented across own certainty conditions. In the uncertain condition, participants received a small amount of clear and easily memorable information, whereas in the certain condition, they were presented with a larger volume of more reliable information at a rapid pace. This creates a potential confound between inferential certainty—where the uncertain condition is indeed less certain—and perceptual certainty—where participants might feel more certain about what they perceived in the uncertain condition, despite its greater ambiguity in interpretation. This distinction could have influenced the neural signals that correlated positively and negatively with our own certainty, potentially accounting for why some of our findings differ from those reported in other neuroimaging studies. Notably, several brain areas, including the anterior insula and dorsolateral PFC, were more active in the certain condition, while they have most commonly been associated with uncertain circumstances (Singer et al. 2009; Badre et al. 2012; Feldmanhall et al. 2019).

A further consideration is the ecological validity of our task design. Even though participants received real peer information, the way this was presented is similar to nonsocial paradigms, in which participants update their decisions based on feedback or new information. Moreover, the social component was somewhat constrained, with participants receiving explicit confidence reports, rather than engaging in interactive social exchanges or interpreting implicit social cues such as in real-world settings (see, for example, Henco et al. 2020; Sevgi et al. 2020). This raises the question of how “social” the task really was. However, our task was modeled closely on the BEAST paradigm (Molleman et al. 2019), which has been used extensively to study social learning and has undergone multiple validation studies. This paradigm has shown that social information use correlates with self-reported conformity and perceived social closeness to the peer, despite minimal contextual information. We therefore belief that our task engages meaningful social processes, even without interaction or rich social cues. In terms of neural results, we did not find peer confidence effects in regions often implicated in social cognition and theory of mind, such as the TPJ or dorsomedial PFC. Previous work has shown that TPJ and dmPFC activation tracked confidence estimates about others’ choices (Trudel et al. 2021; Bang et al. 2022) and revisions of an initial estimate in light of a partner’s estimate (Mahmoodi et al. 2022). One likely explanation is that the level of mentalizing in our task did not depend on reported confidence, leading participants to reflect on each choice equally, regardless of confidence levels. Alternatively, participants might have relied on more general cognitive processes that are not specific to social cognition per se (Ramsey and Ward 2020). Perhaps we would have observed stronger TPJ activation if we had directly compared social and nonsocial sources of information, although this was not the focus of our current research. Importantly, our design did allow for precise control over the reliability of social information, which was critical for isolating the computational mechanisms of social information use.

Finally, our modeling approach was limited to a Bayesian framework, which was grounded in a strong body of previous work suggesting that people and the brain perform Bayesian inference when integrating uncertain information. However, we did not directly compare Bayesian models to alternative model families, such as sequential sampling models. Future work could use model comparison across a broader range of model families to further test whether Bayesian models outperform other frameworks in the context of social information use.

## Conclusion

In conclusion, our work suggests that social information use can be well described by Bayesian principles. Agents take into account the reliability or precision of information, along with a simple heuristic to decide whether to integrate the peer’s information. Finally, our findings suggest that the ventral regions of the mPFC serve as a likely convergence zone for the preparation and execution of integrating precision estimates.

## Supplementary Material

Hofmans_supplementalMaterial_revision_bhaf251

## Data Availability

Data and code will be available through https://osf.io/scywf/.
