# Supplementary material for: Neural correlates of Bayesian social belief updating in the medial prefrontal cortex
Source: Cereb Cortex. 2025 Sep 9;35(8):bhaf251. doi: 10.1093/cercor/bhaf251 (PMC12418961; doi:10.1093/cercor/bhaf251)
Supplement: Hofmans_supplementalMaterial_revision_bhaf251 [file hofmans_supplementalmaterial_revision_bhaf251.docx]

**SUPPLEMENTARY MATERIALS**

Neural correlates of Bayesian social belief updating in the medial prefrontal cortex

**AUTHORS**

Lieke Hofmans^a,b^ and Wouter van den Bos^a,^

^a^ Department of Developmental Psychology, University of Amsterdam, Amsterdam, The Netherlands

^b^ Motivation, Brain and Behaviour Lab, Paris Brain Institute (ICM), Hôpital de la Pitié-Salpêtrière, Paris, France

**CORRESPONDING AUTHOR**

Lieke Hofmans, Hôpital Pitié, 47 Bd de l'Hôpital, 75013 Paris, France

email: [lieke.hofmans@icm-institute.org](mailto:l.hofmans@uva.nl)

**KEYWORDS**

Bayesian decision-making; Computational Modeling; Neuroimaging; Social learning; Uncertainty


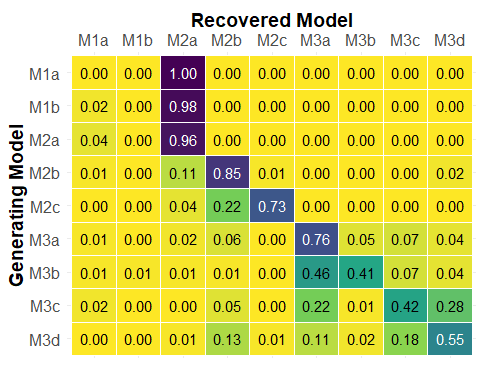


**Figure S1. Model recovery results.** Each model was used to generate simulated data for 169 participants, after which each of these models was fit to these 9 simulated datasets. Model fits were compared using the Bayesian Information Criterion (BIC), and a confusion matrix was constructed to quantify model recoverability. Each entry in this matrix represents the proportion of subjects for whom a given model provided the best fit (lowest BIC) to data generated by each model.

| **Table S1. Parameter recovery: Pearson’s correlations between simulated and fitted parameter values across both experiments.** | | | | | | |
| --- | --- | --- | --- | --- | --- | --- |
|  |  |  |  |  |  |  |
| **M1a** |  |  |  | **M3a** |  |  |
| parameter | *r* | *p* |  | parameter | *r* | *p* |
| α | 0.99 | < 0.001 |  | α_uncertain_ | 0.67 | < 0.001 |
|  |  |  |  | α_certain_ | 0.59 | < 0.001 |
| **M1b** |  |  |  | θ_IC_ | 0.58 | < 0.001 |
| parameter | *r* | *p* |  | θ_slope_ | 0.47 | < 0.001 |
| α_uncertain_ | 0.94 | < 0.001 |  | β | 0.90 | < 0.001 |
| α_certain_ | 0.86 | < 0.001 |  |  |  |  |
|  |  |  |  | **M3b** |  |  |
| **M2a** |  |  |  | parameter | *r* | *p* |
| parameter | *r* | *p* |  | α_uncertain_ | 0.62 | < 0.001 |
| α_uncertain_ | 0.55 | < 0.001 |  | α_certain_ | 0.63 | < 0.001 |
| α_certain_ | 0.53 | < 0.001 |  | θ_IC_ | 0.76 | < 0.001 |
| θ | 0.58 | < 0.001 |  | θ_slope_ | 0.60 | < 0.001 |
|  |  |  |  | β_uncertain_ | 0.87 | < 0.001 |
| **M2b** |  |  |  | β_certain_ | 0.77 | < 0.001 |
| parameter | *r* | *p* |  |  |  |  |
| α_uncertain_ | 0.64 | < 0.001 |  | **M3c** |  |  |
| α_certain_ | 0.67 | < 0.001 |  | parameter | *r* | *p* |
| θ_IC_ | 0.73 | < 0.001 |  | α_uncertain_ | 0.71 | < 0.001 |
| θ_slope_ | 0.74 | < 0.001 |  | α_certain_ | 0.67 | < 0.001 |
|  |  |  |  | θ_IC_ | 0.64 | < 0.001 |
| **M2c** |  |  |  | θ_slope_ | 0.61 | < 0.001 |
| parameter | *r* | *p* |  | β | 0.90 | < 0.001 |
| α_uncertain_ | 0.75 | < 0.001 |  |  |  |  |
| α_certain_ | 0.76 | < 0.001 |  | **M3d** |  |  |
| θ_low_ | 0.75 | < 0.001 |  | parameter | *r* | *p* |
| θ_medium_ | 0.80 | < 0.001 |  | α_uncertain_ | 0.63 | < 0.001 |
| θ_high_ | 0.80 | < 0.001 |  | α_certain_ | 0.68 | < 0.001 |
|  |  |  |  | θ_IC_ | 0.62 | < 0.001 |
|  |  |  |  | θ_slope_ | 0.63 | < 0.001 |
|  |  |  |  | β | 0.96 | < 0.001 |

| **Table S2. Pearson’s correlations between model parameters (M3d) across experiments.** | | | | | |
| --- | --- | --- | --- | --- | --- |
|  |  | $\alpha_{uncertain}$ | $\alpha_{certain}$ | $\theta_{IC}$ | $\theta_{slope}$ |
|  | $\alpha_{uncertain}$ |  |  |  |  |
|  | $\alpha_{certain}$ | 0.62 (*p* < .001) |  |  |  |
|  | $\theta_{IC}$ | 0.58 (*p* < .001) | 0.49 (*p* < .001) |  |  |
|  | $\theta_{slope}$ | 0.28 (*p* < .001) | 0.54 (*p* < .001) | 0.19 (*p* = .012) |  |
|  | $\beta$ | 0.15 (*p* = .054) | 0.03 (*p* = .688) | -0.08 (*p* = .320) | -0.28 (*p* < .001) |
| Note: High correlations between parameter could potentially lead to erroneously fitted values. However, our parameter recovery procedure indicated that these values could be adequately recovered (Table S1). | | | | | |


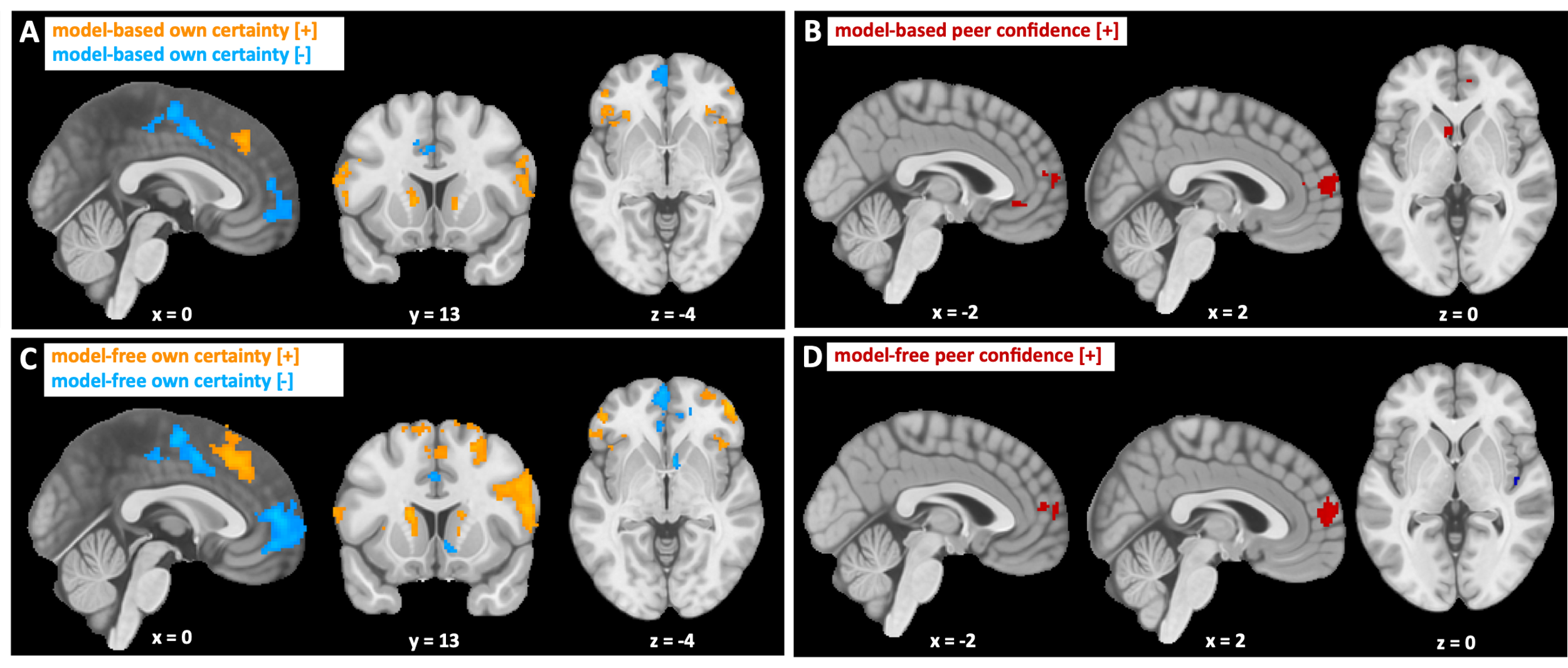
**Figure S2. Neural activity in response to own certainty and peer confidence.** Upper panels depict clusters showing BOLD signal that **(A)** significantly increased (orange) or decreased (blue) in response to *model-based* own certainty or **(B)** significantly increased (red) in response to *model-based* peer confidence. Model-based own certainty and peer confidence are based on the computationally derived parameter values for α and θ, respectively. Lower panels depict clusters showing BOLD signal that **(C)** significantly increased (orange) or decreased (blue) in response to *model-free* own certainty or **(D)** significantly increased (red) in response to *model-free* peer confidence. Model-free own certainty (uncertainty, certain) and peer confidence (low, medium, high) are based on task conditions. Cluster-level corrected, FWE, p < 0.05. Coordinates correspond to MNI space.


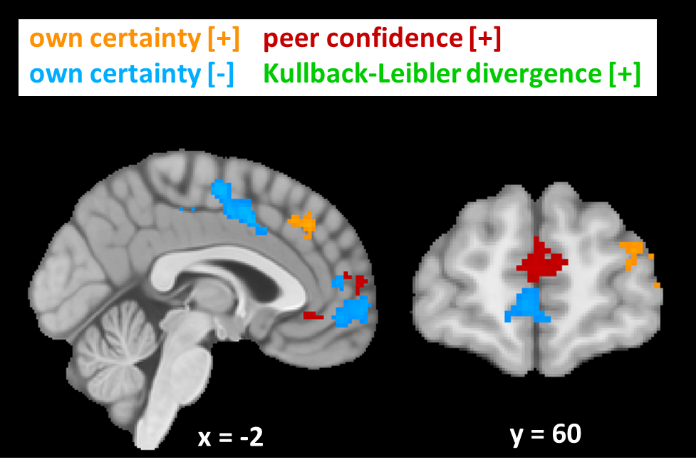


**Figure S3. Neural activity in response to model-based own certainty, peer confidence and Kullback-Leibler divergence when included in the same model.** When KL divergence is added to the GLM with model-based own certainty and peer confidence, the previously found clusters encoding KL divergence are no longer present. This likely reflects shared variance between peer confidence and KL divergence, potentially due to the inherent correlation between the two. Cluster-level corrected, FWE, p < 0.05. Coordinates correspond to MNI space.

NB: In the tables below, sections and areas are based on Glasser MF, Coalson TS, Robinson EC, et al. (2016). A multi modal parcellation of human cerebral cortex. Nature. Nature Publishing Group 536: 171–178.


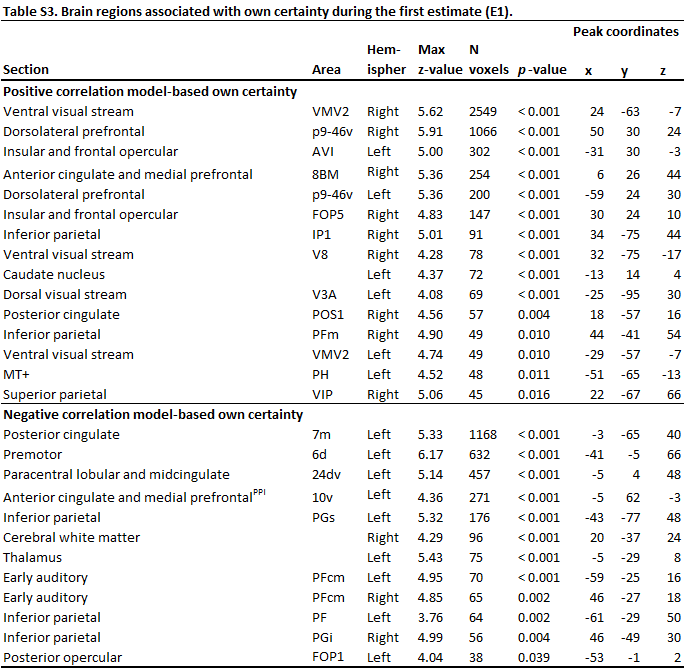

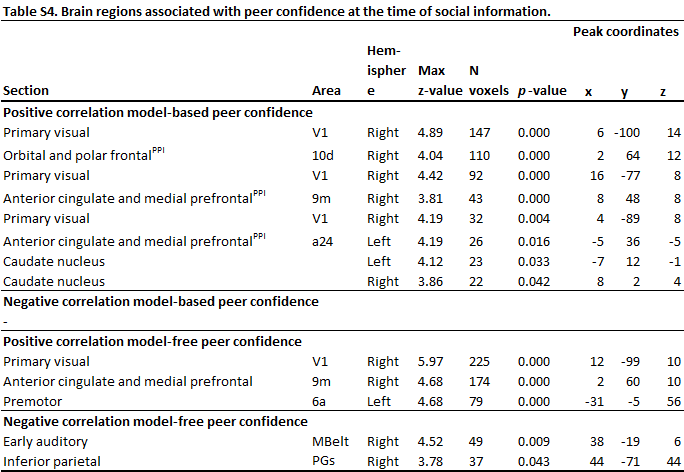

A small cluster in the left premotor cortex showed overlapping activity for two effects: lower stay bias (reflecting a stronger tendency to incorporate peer information) and higher model-free peer confidence. This overlap likely reflects their mathematical relationship, as stay bias is exponentially modulated by model-free peer confidence. Importantly, we did not observe overlapping activity between stay bias and any other neural measures reported in the main text, nor with model-based peer confidence, which is modulated by individually fitted parameter values.
